# Opto-Electronic Hybrid Network Based on Scattering Layers

**DOI:** 10.3390/s23198212

**Published:** 2023-10-01

**Authors:** Jiakang Zhu, Qichang An, Fei Yang, Yuanguo Liu, Yinlong Huo

**Affiliations:** 1 Changchun Institute of Optics, Fine Mechanics and Physics, Chinese Academy of Sciences, Changchun 130033, China; zhujiakang21@mails.ucas.ac.cn (J.Z.); anjj@mail.ustc.edu.cn (Q.A.); liuyuanguo21@mails.ucas.ac.cn (Y.L.); huoyinlong19@mails.ucas.ac.cn (Y.H.); 2 University of Chinese Academy of Sciences, Beijing 100039, China

**Keywords:** scattering layer, opto-electronic hybrid network, target recognition

## Abstract

Owing to the disparity between the computing power and hardware development in electronic neural networks, optical diffraction networks have emerged as crucial technologies for various applications, including target recognition, because of their high speed, low power consumption, and large bandwidth. However, traditional optical diffraction networks and electronic neural networks are limited by long training durations and hardware requirements for complex applications. To overcome these constraints, this paper proposes an innovative opto-electronic hybrid system that combines optical diffraction networks with electronic neural networks. Using scattering layers to replace the diffraction layers in traditional optical diffraction networks, this hybrid system circumvents the challenging training process associated with diffraction layers. Spectral outputs of the optical diffraction network were processed using a simple backpropagation neural network, forming an opto-electronic hybrid network exhibiting exceptional performance with minimal data. For three-class target recognition, this network attains a classification accuracy of 93.3% within a substantially short training time of 9.2 s using only 100 data samples (training: 70 and testing: 30). Furthermore, it demonstrates exceptional insensitivity to position errors in scattering elements, enhancing its robustness. Therefore, the proposed opto-electronic hybrid network presents substantial application prospects in the fields of machine vision, face recognition, and remote sensing.

## 1. Introduction

Advancements in technology, particularly in computing, during the 21st century have propelled humanity toward significant progress in intelligent fields, such as intelligent production, living, and manufacturing. Significant advancements have been achieved in the field of artificial intelligence (AI), which is based on computer technology. Neural networks, supporting target detection, play an indispensable role in machine vision, face recognition, remote sensing, and license plate detection [1,2,3,4]. Increasing demands for efficient neural network technology across various domains has led to the emergence of numerous related techniques.

However, a disparity exists between the development of physical hardware and the practical application requirements, limiting the use of neural networks in computationally intensive applications, such as complex target recognition [5]. To address this limitation, optical neural networks have been proposed as viable alternatives owing to their advantages of low latency, low power consumption, and large bandwidth [6]. These attributes render them suitable for applications where computational resources are constrained. 

However, the implementation of neural networks using optical methods presents challenges in terms of complex hardware infrastructure, leading to increased hardware costs and complexity. To address these challenges, integrating neural networks with optical networks has been considered a new and promising approach.

In traditional integration methods, electronic neural networks are used to train diffraction media, enabling them to acquire the necessary generalization capability for fully optical target classification. This process involves dividing the diffraction media into multiple sub-regions and optimizing the transmittance of each sub-region as a variable. However, this approach introduces a significant number of variables, which in turn slows the optimization process and imposes high hardware requirements. 

To address the challenges presented by the time-consuming training process of diffraction media in conventional optical diffraction networks, this paper proposes a novel method in which the diffraction layers in optical diffraction networks are replaced with fixed scattering media for target preprocessing. By adopting this approach, target discrimination is accomplished through a simple electronic neural network, which not only ensures a sufficient recognition speed but also mitigates the need for high hardware requirements associated with diffraction layers in optical diffraction networks. Artificial neural networks are connection models that emulate the human neural network in information processing and possess robust parallel data processing capabilities. The network comprises numerous nodes that resemble human neurons, responsible for transmitting and processing data. Specific models are constructed by adjusting the weights and biases of neurons in the network. The activation function in the network imparts strong generalization capabilities, rendering it highly suitable for applications such as target recognition.

Different types of neural networks offer broad prospects in various applications, such as target recognition, classification, autonomous driving, translation, and clinical medicine [7,8,9,10,11,12]. In the field of optics, the continuous advancement of neural networks has led to the development of new optical technologies. These developments present significant possibilities for further improvements and innovation in the field. 

The integration of neural networks with optical technologies is a significant milestone in the field of optics, resulting in intelligent optics. This advancement has notably improved the bandwidth-related performance in wave-front detection and prediction [13,14,15], infusing new vitality into related fields. However, for the practical applications of neural networks, their ability to process scene variability is crucial, challenging their generalization and parallel computing capabilities.

Currently, various approaches, including increasing training data, regularization, and convex optimization [16], are employed to enhance the generalization ability of the networks. However, they lead to an increase in the data volume, posing potential structural challenges in the network. Achieving a balance between the enhancement of generalization and the maintenance of the network efficiency remains a key consideration for practical implementation. As data volume increases, the need for additional nodes and layers for data processing increases accordingly. However, this expansion leads to a heavy computing load, which, in turn, decreases the processing speed and leads to new demands regarding the network bandwidth. Consequently, in electronic platform-based neural networks, the growing bandwidth requirements of the network and electronic hardware development gradually become mismatched owing to physical hardware limitations. This results in a significant widening of the gap between these two aspects over the years. 

Optical computing methods have extensive applications in various fields, including data storage and image recognition, owing to their numerous advantages, such as low latency, large bandwidth, low power consumption, and robust parallel processing capabilities [17,18,19]. These methods can be broadly categorized into two main types: analog and digital optical computing.

Digital optical computing primarily relies on optical logic gates, whereas analog optical computing constructs networks through matrix multiplication and nonlinear functions [20]. The realization of neural networks using optical methods represents a new and promising direction that has emerged because of the continuous development of neural networks. This innovative approach presents new possibilities and potential advancements in the field of optical computing and its integration with neural networks. This approach inherits the potent nonlinear fitting capability of neural networks while benefiting from the low latency and large bandwidth characteristics in optics, offering an efficient method to address the challenge of balancing extensive computation requirements with the physical structure development of electronic platforms [21,22]. However, when compared with electronic platforms, photonic platforms are more complex in terms of hardware and generally exhibit lower accuracy in certain applications, such as target recognition, creating a significant gap between this technology and electronic neural networks. To bridge this gap, the introduction of a full optical diffraction network that connects neural networks with optical networks has been proposed [23]. 

Through the training of multiple layers of diffraction media using neural networks, the method achieves non-linear mapping effects [24,25], enabling comprehensive optical target classification. The approach involves employing the optical backpropagation algorithm [26] to continuously update the transmittance of each diffraction element in the diffraction media during the training process. Increasing the number of diffraction elements and diffraction layers, along with the implementation of differential algorithms, can lead to improved target classification accuracy in complex target classification scenarios [27]. 

The generalization ability of optical diffraction networks is closely related to the number of diffraction elements, necessitating a large number of such elements to achieve rapid target classification at the speed of light. However, this leads to a significant time requirement and high hardware demands during the training of diffraction layers, particularly in multi-layer diffraction networks.

Scattering, a common phenomenon in daily life, is often considered an error source owing to its distortion and attenuation effects on incident light in practical applications. In 2010, an article on super-resolution imaging triggered a surge in research on scattering media [28], leading to increased interest and exploration in the field. 

Scattering media currently have extensive applications in various fields, such as super-resolution imaging, multispectral imaging, and non-line-of-sight imaging [29,30], exhibiting its strong control over the optical field as a disordered medium. To overcome the drawbacks of long training durations and high hardware requirements in purely optical diffraction networks, this paper introduces an opto-electronic hybrid system. In the proposed method, the diffraction layers in optical diffraction networks are replaced with scattering media that do not require pre-training.

Benefitting from the powerful generalization capability of scattering media for the white light input, this method enables efficient target preprocessing. The resulting spectral data are then processed using a simple electronic neural network, enabling rapid and accurate target discrimination. This opto-electronic hybrid strategy combines the strengths of scattering media and electronic neural networks, offering a promising method to address the challenges associated with traditional optical diffraction networks. 

In this opto-electronic hybrid network framework, fixed scattering layers were used to effectively replace the diffraction layers, eliminating the need for long training durations and high hardware requirements. Owing to the significant differences between spectral data of different target categories, accurate classification can be achieved using a simple neural network. This results in the realization of a near-light processing speed, similar to that of traditional optical diffraction networks, with modest hardware configurations.

**Figure 1:**
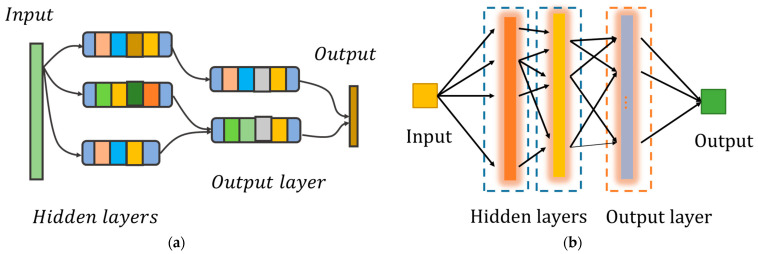
Overall structure of the optical diffraction network based on scattering layers. (**a**) Traditional CNN: The different small squares of the hidden layer represent the structures of the convolutional neural network, such as the convolutional layer and the pooling layer, which take a long time, and mainly play a role in feature extraction. The output layer is considered a classifier. (**b**) Photoelectric hybrid network based on scattering layer: The hidden layer is composed of optical diffraction network composed of scattering layer and micro-lens array, while the output layer is composed of electrical network to complete the classification judgment.

Furthermore, unlike traditional convolutional neural networks (CNNs) that require a lot of time for operations such as convolution and pooling during training, the optical network part of this structure can perform target preprocessing at the speed of light, thereby shortening the overall training time of the network. The adoption of simple electronic components further contributes to rapid target recognition, as illustrated in Figure 1. Therefore, this structure combines the advantages of high-speed optical diffraction networks with low power consumption and large bandwidth, achieving target classification through a simple electronic network.

## 2. Principles

**Figure 2:**
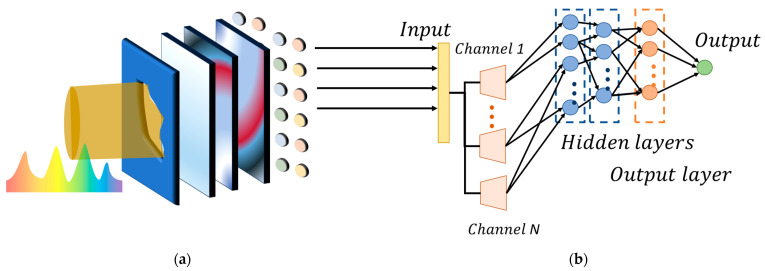
Photoelectric hybrid network model based on scattering layer. (**a**) Optical diffraction model, with the scattering layer replacing the diffraction layer. Polychromatic light passes through the aperture stop, target, micro-lens array, two layers of scattering media, and focusing lens and is finally received by the spectrometer. There is no strict limit to the axial distance between the different components, which is specified in Section 3. (**b**) Electrical neural network model. We use a simple three-layer backpropagation neural network to process the received spectrum of the spectrometer, thus achieving rapid judgment of the target class.

In the optical diffraction network configuration, two layers of scattering media and one layer of micro-lens array are utilized. The introduction of the micro-lens array, instead of the scattering layer, allows the incident light to be divided into different parts, enhancing the generalization capability of the optical diffraction network and leading to a high classification accuracy. The configuration is illustrated in Figure 2a.

The network is directly constructed using the existing scattering layer. Moreover, the scalar diffraction theory is employed to describe the transmission of complex amplitudes between different components in the optical diffraction model. This theory provides a rapid and accurate method for obtaining theoretical results while simplifying the transmission process of light in the system.

In an optical neural network, the incident polychromatic light $E_{stop\_in}$ at the aperture stop can be represented as follows:

(1) $$E_{stop\_in}\left(x_{1},y_{1},\lambda \right)=a\left(x_{1},y_{1},\lambda \right)\exp\left(j\phi \left(x_{1},y_{1},\lambda \right)\right),$$

where $\;(x_{1},y_{1})$ is the plane of the aperture stop;$\;a\left(x_{1},y_{1},\lambda \right)$ is the amplitude of the incident light; j is the imaginary unit; and $\;\phi \left(x_{1},y_{1},\lambda \right)$ is the phase of the incident light. Here, the incident light is assumed as a plane wave propagating perpendicular to the optical axis. Consequently, $\phi \left(x_{1},y_{1},\lambda \right)=0$. Therefore, the incident light at the aperture stop can be expressed as follows:

(2) $$E_{stop\_in}\left(x_{1},y_{1},\lambda \right)=a\left(x_{1},y_{1},\lambda \right)$$

The complex amplitude transmittance function of the circular aperture stop can be represented as follows:

(3) $$t_{stop}\left(x_{1},y_{1}\right)=circ\left(\frac{r}{a}\right),$$

where $r=\sqrt{x_{1}^{2}+y_{1}^{2}}$ and a is the radius of the circular aperture stop. Therefore, the complex amplitude behind the aperture stop can be expressed as follows:

(4) $$E_{stop\_out}\left(x_{1},y_{1},\lambda \right)=a\left(x_{1},y_{1},\lambda \right)\;t_{stop}\left(x_{1},y_{1}\right)=a\left(x_{1},y_{1},\lambda \right)\;circ(\frac{r}{a})$$

The propagation of light between different elements follows the scalar diffraction theory. Therefore, the complex amplitude distribution in front of the target can be written as follows:

(5) $$\begin{array}{c} E_{target\_in}\left(x_{2},y_{2},\lambda \right)=\frac{\exp\left(jk\lambda \right)}{jkz_{12}}exp(j\frac{k}{2z_{12}}\left(x_{2}^{2}+y_{2}^{2}\right))\psi [E_{stop\_out}\left(x_{1},y_{1},\lambda \right)\\ exp(j\frac{k}{2z_{12}}\left(x_{1}^{2}+y_{1}^{2}\right))]{|}_{f_{x2}=\frac{x_{2}}{\lambda z_{12}},f_{y2}=\frac{y_{2}}{\lambda z_{12}}}, \end{array}$$

where $\left(x_{2},y_{2}\right)$ is the plane of the target; $z_{12}$ is the horizontal distance along the optical axis between the aperture stop and the target; and ψ is the Fourier transform. The complex amplitude distribution $E_{MLA\_in}$ in front of the micro-lens array can be expressed as follows:

(6) $$\begin{array}{c} E_{MLA\_in}\left(x_{3},y_{3},\lambda \right)=\frac{\exp\left(jk\lambda \right)}{jkz_{23}}exp(j\frac{k}{2z_{23}}\left(x_{3}^{2}+y_{3}^{2}\right))\psi [E_{target\_in}\left(x_{2},y_{2},\lambda \right)t_{target}(x_{2},y_{2})\\ exp(j\frac{k}{2z_{23}}\left(x_{2}^{2}+y_{2}^{2}\right))]{|}_{f_{x3}=\frac{x_{3}}{\lambda z_{23}},f_{y3}=\frac{y_{3}}{\lambda z_{23}}}, \end{array}$$

where ${(x}_{3},y_{3})$ is the plane of the micro-lens array;${\;z}_{23}$ is the distance between the target and the micro-lens array; and $t_{target}(x_{2},y_{2})$ is the complex amplitude transmittance function of the target. The complex amplitude $E_{layer1\_in}$ in front of the scattering layer can be expressed as follows:

(7) $$\begin{array}{c} E_{layer1\_in}\left(x_{4},y_{4},\lambda \right)=\frac{\exp\left(jk\lambda \right)}{jkz_{34}}exp(j\frac{k}{2z_{34}}\left(x_{4}^{2}+y_{4}^{2}\right))\psi [E_{MLA\_in}\left(x_{3},y_{3},\lambda \right)t_{MLA}(x_{3},y_{3})\\ exp(j\frac{k}{2z_{34}}\left(x_{3}^{2}+y_{3}^{2}\right))]{|}_{f_{x4}=\frac{x_{4}}{\lambda z_{34}},f_{y4}=\frac{y_{4}}{\lambda z_{34}}}, \end{array}$$

where $\left(x_{4},y_{4}\right)$ is the plane of scattering layer 1; $z_{34}$ is the distance between the micro-lens array and scattering layer 1; and $t_{MLA}(x_{3},y_{3})$ is the complex amplitude transmittance function of the micro-lens array, which can be expressed as follows:

(8) $$t_{MLA}\left(x_{3},y_{3},\lambda \right)=\sum_{i=1}^{N}\sum_{j=1}^{M}\delta \left(x_{3}-id,y_{3}-jd\right)⨂exp(-j\frac{k}{2f}\left(x_{3}^{2}+y_{3}^{2}\right)),$$

where N and M are the number of micro-lenses in the $\;x_{3}$ and $\;y_{3}$ directions, respectively; d is the period of the micro-lens array; f is the focal length of the micro-lens array; and ⨂ is the symbol representing convolution operation. 

When light propagates through a scattering medium, it scatters both forward and backward. In the case of a relatively thick scattering medium, multiple scatterings can occur, rendering it challenging to accurately represent light propagation with a single model. In speckle imaging and related fields, the memory effect of the scattering medium is a critical research focus. However, this memory effect is limited and inversely proportional to the thickness of the medium, which hinders the application of methods such as the cross-correlation method in speckle imaging. Numerous studies are currently exploring various methods to enhance the memory effect range of the scattering medium [31,32,33]; however, the mitigation of the aforementioned issue remains challenging.

In addition to the memory effect, the transmission matrix of the scattering medium is a significant research focus [34,35,36]. The determination of the transmission matrix of the scattering medium is crucial for obtaining the control effect of the incident light field. This issue constitutes the core focus of this research, with an emphasis on time-varying and non-uniform scattering media, which are associated with significant challenges. Let T_1 and T_2 represent the transmission matrices of scattering layer 1 and scattering layer 2, respectively. Accordingly, the complex amplitude distribution in front of scattering layer 2 can be written as follows:

(9) $$\begin{array}{c} E_{layer2\_in}\left(x_{5},y_{5},\lambda \right)=\frac{\exp\left(jk\lambda \right)}{jkz_{45}}exp(j\frac{k}{2z_{45}}\left(x_{5}^{2}+y_{5}^{2}\right))\psi [E_{layer1\_in}\left(x_{4},y_{4},\lambda \right)T_{1}(x_{4},y_{4},\lambda )\\ exp(j\frac{k}{2z_{45}}\left(x_{4}^{2}+y_{4}^{2}\right))]{|}_{f_{x5}=\frac{x_{5}}{\lambda z_{45}},f_{y5}=\frac{y_{5}}{\lambda z_{45}}} \end{array}$$

where $\left(x_{5},y_{5}\right)$ is the plane of scattering layer 2, and $z_{45}$ is the axial distance from scattering layer 1 to scattering layer 2. The incident complex amplitude in front of the focusing lens can be written as follows:

(10) $$\begin{array}{c} E_{lens\_in}\left(x_{6},y_{6},\lambda \right)=\frac{\exp\left(jk\lambda \right)}{jkz_{56}}exp(j\frac{k}{2z_{56}}\left(x_{6}^{2}+y_{6}^{2}\right))\psi [E_{layer2\_in}\left(x_{5},y_{5},\lambda \right)T_{2}(x_{5},y_{5},\lambda )\\ exp(j\frac{k}{2z_{56}}\left(x_{5}^{2}+y_{5}^{2}\right))]{|}_{f_{x6}=\frac{x_{6}}{\lambda z_{56}},f_{y6}=\frac{y_{6}}{\lambda z_{56}}} \end{array}$$

where $\left(x_{6},y_{6}\right)$ represents the lens where the focusing plane is located, and $z_{56}$ is the axial distance between scattering layer 2 and the focusing lens. Finally, the received complex amplitude at the spectrometer can be represented as follows:

(11) $$E_{S}\left(x_{7},y_{7},\lambda \right)=\frac{\exp\left(jk\lambda \right)}{jkz_{67}}\exp\left(j\frac{k}{2z_{67}}\left(x_{7}^{2}+y_{7}^{2}\right)\right)\psi \left[E_{lens_{in}}\left(x_{6},y_{6},\lambda \right)\right]{|}_{f_{x7}=\frac{x_{7}}{\lambda z_{67}},f_{y7}=\frac{y_{7}}{\lambda z_{67}}},$$

where $\left(x_{7},y_{7}\right)$ represents the plane of the spectrometer receiver, and $z_{67}$ is the axial distance between the focusing lens and spectrometer receiver. Generally, this distance is equal to the focal length of the focusing lens. After receiving the spectral data from the target, the spectrometer inputs the data into an electronic neural network to obtain the category of the target.

During the training process of this opto-electronic hybrid network, the optical diffraction network, utilizing the scattering layer, can generate spectral output data with significant variations, enabling rapid target classification without an extensive amount of data. Therefore, the use of scattering layers in the optical diffraction network can reduce the demand for electronic hardware of the network in complex applications.

Unlike convolutional neural networks that can adjust parameters in real time during training, once the optical part of this opto-electronic hybrid network determines the scattering layer, the relevant parameters cannot be changed. However, employing scattering layers and micro-lens arrays facilitates maximizing the values of parameters that can be adjusted, similar to those achieved using a CNN. Even with a fixed structure, the optical diffraction network possesses adequate generalization capability for target recognition in complex scenarios, operating at the speed of light.

In contrast, data processing stages, such as the electronic neural network, involve discrete data flow, which inevitably introduces the issue of discrete data sampling. The higher complexity or precision of desired functionality often requires a higher level of sampling or more parameters. However, this issue does not arise in practical optical paths, as operations within optical paths are continuous. Consequently, in contrast to the discrete transmittance during the training process of the traditional optical diffraction network, using the electronic neural network, the replacement of the diffraction layer with scattering media, allows target classification based on its inherent properties. Despite its powerful generalization capability, this approach may not yield definitive results, necessitating the electronic neural network to provide a definite classification outcome. Benefitting from the potent target feature extraction capability of the optical diffraction network, this study aims to use a simple three-layer backpropagation neural network (BPNN) to fulfill the role of the classifier (Figure 2b). 

## 3. Results

### 3.1. Experimental Setup

**Figure 3:**
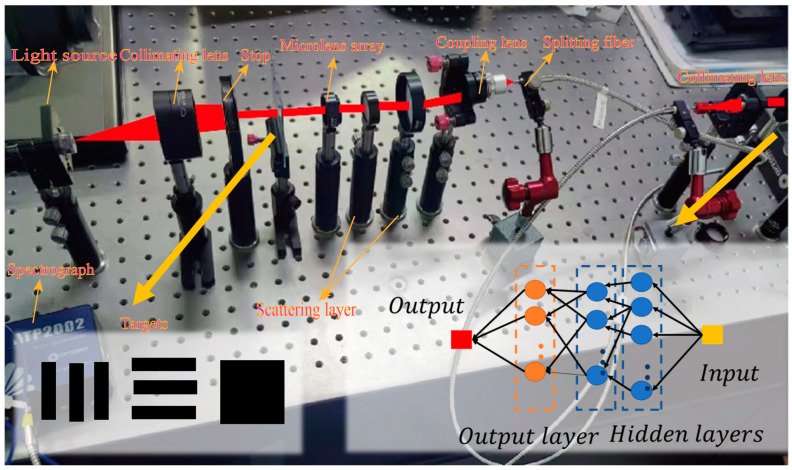
Schematic of the experimental setup. In the lower left corner are the three main objects involved in the experiment: vertical line, horizontal line, and square. They are all provided by the same optical calibration board. After the coupling lens couples the light field, it is divided into two ways through the beam splitter, one input to the spectrometer, the other input to the CCD, to detect whether the spectrometer receives the light field from the target.

The composition of the entire experimental setup includes: an incoherent light source, two collimating lenses, an aperture stop, an optical resolution plate, a micro-lens array, two scattering layers, a coupling lens, a splitting fiber, a CCD, and a spectrometer, as shown in Figure 3. The light source is a halogen lamp used to generate broad-spectrum incident light. The focal length of the collimating lens is 100 mm, used to collimate the broad-spectrum light emitted by the light source. The distance between the first collimating lens and the light source is approximately equal to its focal length. The light collimated after passing through the collimating lens is modulated by a circular aperture stop. The size of the aperture stop is adjusted according to the target on the optical resolution plate to prevent stray light from entering the system. The micro-lens array is placed behind the aperture stop. It has dimensions of 30 × 30, a diameter of 300 um, and a focal length of 14.6 mm. The presence of the micro-lens array not only improves optical energy utilization, as can be seen in the following sections, but also divides the spatial light with target information into different parts, enhancing the nonlinear mapping capability of the optical diffraction network. The scattering layer is the core of the optical diffraction network, mapping the spatial light with different targets into different spectral information, completing the preliminary preprocessing step. The coupling lens couples the light field behind the scattering layer into the splitting fiber. The splitting fiber divides the light into two paths. One path passes through a collimating lens and is received by a CCD to monitor whether the spectrometer receives the light field from the target, while the other path is directly connected to the spectrometer, outputting spectral data. There are no strict restrictions on the distance and relative positions of the components; the key is to ensure that the light field behind the scattering layer can be coupled into the splitting fiber.

To verify the specific application potential of the proposed structure, three experiments were designed. In the first experiment, two targets with the same area but different shapes were used as inputs to investigate whether the variations in received spectra were caused by different target intensities. The experiment also analyzed the influence of the relative positions of the scattering elements on the output results. 

In the second experiment, input targets consisting of squares, horizontal lines, and vertical lines from the same row of the calibration board were utilized. Variations in the input targets were achieved by altering relative distances or rotating the targets. For each target, 100 spectral data points were collected to construct a simple electronic neural network for basic target classification.

In the third experiment, transparent thin plates were used to replace the targets, and a randomly perturbed tape was attached to simulate scenes with and without turbulence. The goal of this experiment was to verify the potential application of the proposed structure in classifying the turbulence intensity.

In terms of acquiring training data, we used three patterns on the optical resolution plate: horizontal lines, vertical lines, and squares, as classification targets. During the collection of training data for each category of targets, the target is changed by changing the relative position of the optical resolution plate. Based on this approach, we collected 100 spectral data points for each category of targets, with 70 of them used for the training set, and the remaining 30 for the test set.

### 3.2. Influence of Equal-Area Targets and Relative Positions of the Scattering Layer

In the optical diffraction network, using patterns from the optical calibration board as inputs may result in varying target areas in many cases. As a result, the generalization ability of the optical diffraction network might not solely originate from the nonlinear mapping of the input wavelength intensity by the network. The generalization capability of the network could also be affected by the different target areas, introducing ambiguity in the mapping object of the network itself. To address this issue, two input targets, one square and one triangle, were employed. These targets with different shapes have the same area. In addition, we also analyzed the output spectra of targets with different areas and the same shape.

**Figure 4:**
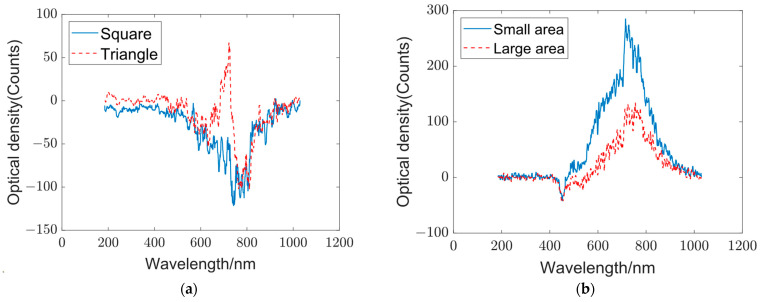
Optical diffraction network output for equal target areas and same target shape. (**a**) Output spectra of targets with the same area but different shapes: The blue curve represents the output spectrum of the square target, whereas the red curve represents the output spectrum of the triangle target. (**b**) Output spectra of targets with different areas and the same shape. The blue curve represents the output spectrum of the target with a small area, and the red curve represents the output spectrum with a large area.

We used squares and triangles with the same area but different shapes to verify the influence of shape on the output spectrum of the optical diffraction network, and we also used two horizontal lines with the same shape but different areas to verify the influence of area on the output spectrum. The spectral data are displayed in Figure 4. When considering equal target areas (Figure 4a), the output spectra of both targets appear similar for wavelengths below 600 nm and above 800 nm. However, in the spectral range between 600 and 800 nm, the triangle target spectrum exhibits a peak, whereas the square target spectrum shows a decreasing trend. Consequently, the generalization ability of the optical diffraction network does not solely originate from variations in target areas. Instead, it arises from the diverse mappings of different wavelengths at different positions due to the presence of scattering media. When considering the same shape, the output spectra of vertical targets under different areas have strong similarity, as shown in Figure 4b. This means that this scattering layer-based optical diffraction network is sensitive to shape changes, while changes in area only scale the output spectrum accordingly, which has no impact on classification.

In traditional optical diffraction networks, the relative position error of the diffraction layer can be introduced during training to compensate for the decrease in network classification accuracy caused by assembly errors in the actual hardware structure [24]. This indicates that the relative positions of the diffraction layer in traditional optical diffraction networks impact the accuracy of target classification to a certain extent. Therefore, it needs to be further determined whether the relative position of the scattering layer affects the output spectrum in our proposed structure.

In the experiment, changes in the output spectra were examined by adjusting the position and rotation angle of the second scattering layer. The axial displacement ∆z was varied within ±10 mm, and the rotation angle ∆θ was varied from 0° to 30°. The output spectra at the initial, maximum, and minimum positions were compared.

**Figure 5:**
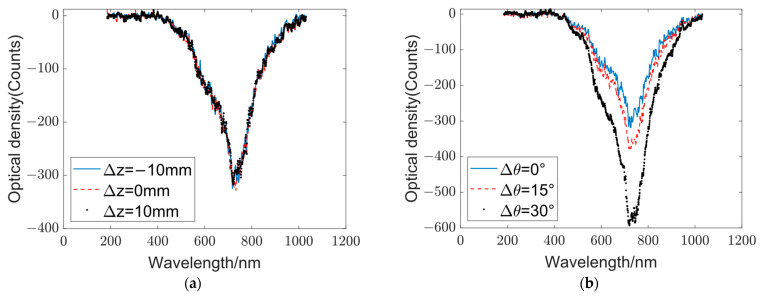
Influence of relative positions of the scattering layers. (**a**) Output spectra at varying axial distances: the blue, red, and black curves represent the output spectra at Δz = −10, 0, and 10 mm, respectively. (**b**) Output spectra at changing angles: the blue, red, and black curves represent the output spectra at Δθ = 0°, 15°, and 30°, respectively.

When the axial distances between the scattering layers change continuously, no significant alterations are observed in the output spectra, as illustrated in Figure 5b. However, as the angle of the scattering layer gradually increases from 0° to 30°, the peak in 670 nm of the output spectra also increases, whereas the overall shape remains similar, as shown in Figure 5b. 

A possible reason for this outcome is that the output of the optical diffraction network is a spectrum, and the impact of the axial displacement between the diffraction layers on the output spectrum is substantially smaller compared with the influence of the scattering layers themselves. In this context, the spectral changes caused by the axial displacement between the diffraction layers are obliterated by the effect of the scattering layers on the output spectrum. When the angle of the scattering layer changes, the impact on the output spectrum exhibits a linear relationship. Consequently, the shape of the output spectrum remains consistent even with changes in the angle of the scattering layer.

In the optical diffraction network, different targets yield different output spectra, and the subsequent electronic neural network determines the target categories based on these distinct output spectra. When the relative positions of the optical network change, the output spectra change proportionally; however, the spectral shape remains unchanged. Therefore, the optical diffraction network is not sensitive to the position errors of the scattering layers and other components, which is advantageous for installation and adjustment.

### 3.3. Target Recognition Using the Opto-Electronic Hybrid Network

In traditional optical diffraction networks, 3D printing technology or other methods can be used to convert the diffraction layers trained by the electrical network into actual hardware, and then build the actual optical path. After obtaining result data, the target category can be determined by processing the results with differential algorithms. However, when the diffraction layer is replaced with a scattering layer, the generalization ability provided by the scattering layer becomes less deterministic. As a result, an electronic neural network is required to act as a classifier for target classification during data processing, forming the fundamental concept of using opto-electronic hybrid networks for target recognition.

**Figure 6:**
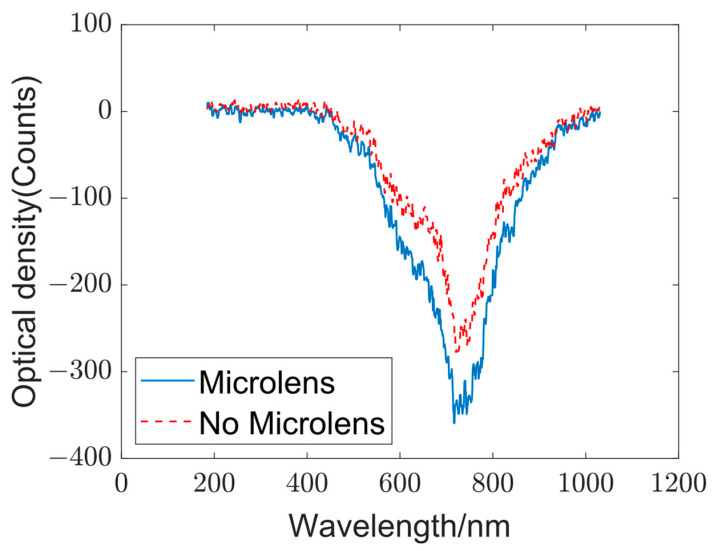
Influence of micro-lens array on the output spectra: The blue and red curves represent the output spectra with and without the micro-lens array, respectively.

In the optical diffraction network, a micro-lens array is incorporated to partition the incident light into different segments, enhancing the nonlinearity of the network. In the experiment, the output spectra with and without the micro-lens array were compared to determine whether introducing the micro-lens array in the optical diffraction network could increase the level of differentiation in the output spectra and improve the accuracy of target classification. The output spectra are presented in Figure 6.

Although the output spectra with and without the micro-lens array exhibit similar shapes, the intensity of the output spectra obtained with the micro-lens array is higher than that acquired without it. Notably, clear turning points are observed in the spectra at approximately 650, 800, and 850 nm. The increased intensity of the spectra obtained with the micro-lens array is primarily attributed to the focusing effect of the micro-lens array, which effectively harnesses the input light energy. As a result, the opto-electronic network with a micro-lens array enhances differentiation in the output spectra while improving the utilization of light energy. This approach effectively enhances the accuracy of classification in target recognition and other relevant fields. 

**Figure 7:**
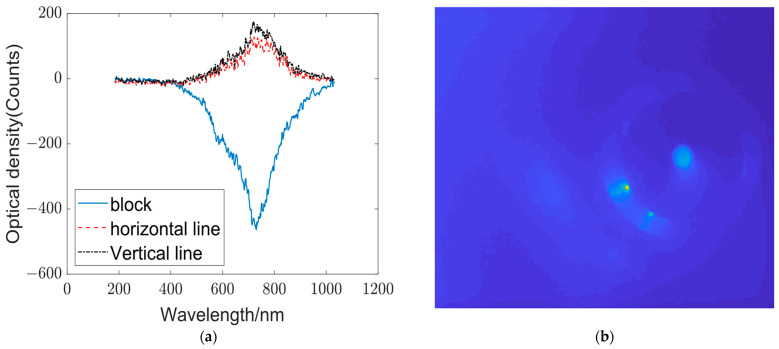
Output spectra of the three types of targets. (**a**) Comparison of the output spectra of the three types of targets: The blue, red, and black curves represent the output spectra of the square, horizontal line, and vertical line, respectively. (**b**) Image received by the CCD.

After confirming that the introduction of the micro-lens array can improve the nonlinear mapping ability of the network, we built a complete optical diffraction network, and established the data set according to the data acquisition process described in Section 3.1. The electronic neural network is then utilized to perform the classifier function for target recognition. In the experiments, a three-class opto-electronic hybrid network was implemented for square, horizontal line, and vertical line classification (Figure 3). Changes in targets were achieved by moving and rotating the optical calibration board. The spectral data for the three categories are displayed in Figure 7a.

Notably, the output spectra of squares differ significantly from those of the other targets, whereas the output spectra of horizontal lines and vertical lines are similar but exhibit certain variations in the peak intensity and corresponding wavelengths. A comparison of the output spectra of the three categories in the same graph clearly reveals the distinctions between the spectra of different targets.

During spectral reception, a CCD camera was employed to monitor the reception status of the spectral end, as shown in Figure 7b. The presence of a spot image in the CCD indicates that the spectral input has passed through the target.

**Figure 8:**
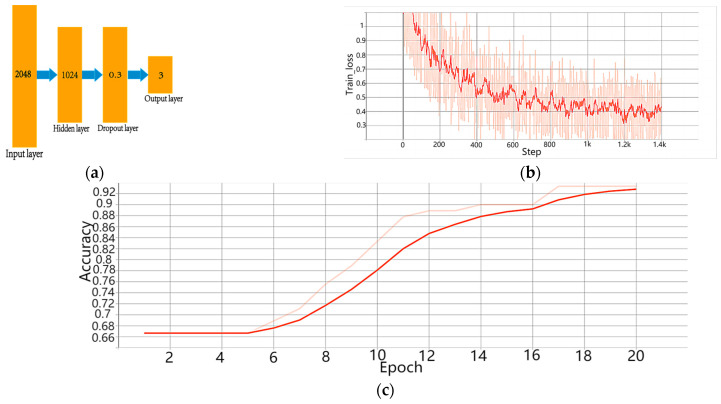
Electrical neural network classifier. (**a**) Three-layer BPNN structure: A network structure with 2048 inputs and 3 outputs. Hidden layer has 1024 nodes, and a Dropout layer with a probability of 0.3 is used. (**b**) Loss function of the training set. The light red curve represents the actual result, while the red curve represents the fitted result. (**c**) Accuracy of the test set. The light red curve represents the actual result, while the red curve represents the fitted result.

For implementing the classifier, a three-layer BPNN was employed, as illustrated in Figure 8a. The input comprised 2048 discrete spectral data; the hidden layer had 1024 nodes; and a Dropout layer with a probability of 0.3 was introduced after the middle layer to prevent overfitting. The output layer consisted of three nodes, each corresponding to one of the three target categories. The training results are displayed in Figure 8b,c.

Utilizing the opto-electronic hybrid network with scattering layers, an appreciable classification accuracy of 93.3% was achieved for the three target categories. Notably, when compared with traditional CNNs and optical diffraction networks, the proposed network exhibited a slightly lower training accuracy. This discrepancy could be attributed to the similarity between the horizontal and vertical lines used in the experiment. The limited number of samples for these two targets resulted in a decreased accuracy in distinguishing them effectively.

The use of the proposed opto-electronic hybrid network structure eliminates the time-consuming convolution and pooling operations in traditional electronic CNNs via optical diffraction networks, resulting in a significantly reduced training time and less sophisticated hardware requirements during training. A comparison of the traditional electronic CNN, traditional optical diffraction network, and improved opto-electronic hybrid network (Table 1) demonstrates that the opto-electronic hybrid network structure achieves rapid training and high accuracy even under suboptimal hardware conditions.

### 3.4. Application Potential of Opto-Electronic Hybrid Networks in Turbulent Scenes

In certain applications, the optical path exposed to air is often affected by atmospheric turbulence, which can adversely impact precision measurements, geodesy, and guidance operations. Hence, assessing the strength of turbulence in the measurement area is necessary to guide relevant practical operations. Traditional methods for measuring the turbulence intensity rely on meteorological parameters, beam propagation, laser radar, and others, but they either have high costs or involve complex data processing.

To address these challenges, a new approach is essential for simple turbulence intensity measurement. The emergence of neural networks has revitalized various fields, including turbulence measurement, leveraging big data in their applications. To verify the potential application of the proposed opto-electronic hybrid network in measuring the turbulence intensity, relevant experiments were conducted to distinguish between scenes with and without turbulence.

**Figure 9:**
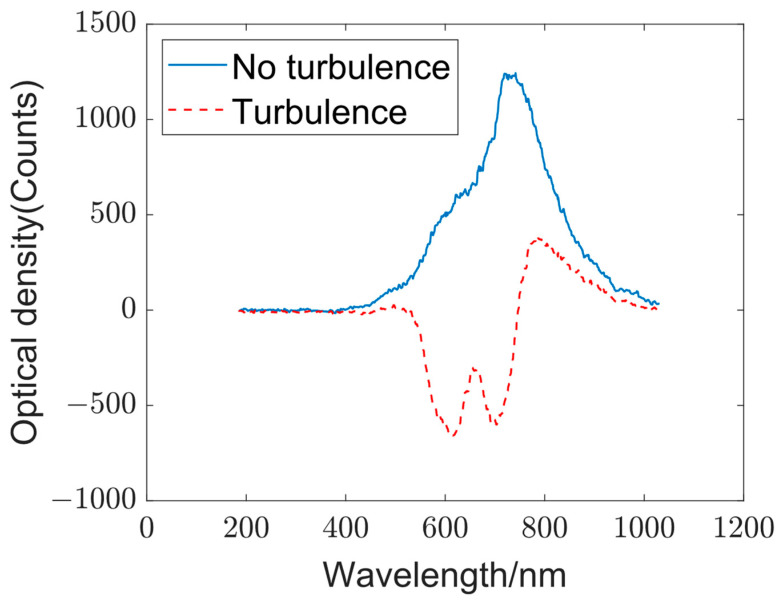
Output spectra for scenes with and without turbulence. The blue curve represents the output spectrum in the absence of turbulence, whereas the red curve represents the output spectrum in a turbulent scene.

The transparent region of the optical calibration board represents a turbulence-free scene, whereas the area covered with blue tape indicates the turbulent region (Figure 3a). Figure 9 displays the output spectra of the two scenes. An evident distinction in output spectra between scenes with and without turbulence was observed, confirming the high differentiation capability of the opto-electronic hybrid network. However, the simulation of scenes considered herein is simplified, and practical scenarios may introduce numerous uncertainties. Consequently, the next step involves the use of a phase screen to quantify the turbulence intensity and evaluation of the practical applicability of the network under real-world condition.

## 4. Discussion

To overcome the drawbacks of traditional optical diffraction networks and electronic neural networks, which involve high hardware requirements and long training durations, this study proposes an innovative opto-electronic hybrid network. In this architecture, the pre-training requirement of the diffraction layer in traditional optical diffraction networks is circumvented by replacing it with a scattering layer. The electronic neural network acts as a classifier for target classification.

In opto-electronic hybrid networks, it can be considered that time-consuming operations such as convolution and pooling in traditional convolutional neural networks are achieved at the speed of light. This integration enables the rapid extraction of target features, while the retained simpler components effectively serve as a classifier for target classification. 

This opto-electronic network architecture achieves network training with lower hardware configurations and requires fewer data than the traditional networks, while retaining the advantages of traditional optical diffraction networks, such as a high speed, low power consumption, and large bandwidth. Before constructing the network, output spectra from two targets with equal areas but different shapes were compared. The findings revealed that the generalization ability of the opto-electronic hybrid network is not solely dependent on different target areas but rather on the distinct mappings of various wavelengths from different parts of the target by the scattering layer.

Subsequent experiments were conducted to confirm the performance of this opto-electronic hybrid network. In a three-class application with similar targets, the network achieved a significant training time of 9.2 s and an appreciable accuracy of 93.3% when utilizing a CPU I5-7300H and GPU RTX 1050Ti. These results were obtained with 100 data points (70 for training and 30 for testing) collected for each target. This approach effectively reduces the training time while easing hardware requirements. For the improved optical diffraction network, the influence of the relative positions of the scattering layers on the output spectra was extensively investigated. The results indicated that changes in the axial displacement did not significantly affect the output spectra, whereas changes in the angle of the scattering layers had a linear impact on the output spectra. This insensitivity to the relative positions of the scattering layers in the optical diffraction network is advantageous for optical alignment. However, it also suggests that enhancing the generalization ability of the network through adjustments in the positions of the scattering layers is not feasible. Instead, the generalization ability can be improved by increasing the number of scattering layers or utilizing highly complex scattering layers. Finally, the potential application of the proposed opto-electronic hybrid network in classifying the turbulence intensity was demonstrated through experiments. Although uncertainties exist in the experimental setup, such as those related to environmental conditions, the experiment provides a basis for verifying the performance of the proposed architecture in turbulence intensity classification using a phase screen.

## 5. Conclusions

In this paper, we designed an opto-electronic hybrid network based on scattering layers and verified its performance using a three-target classification task. In the optical diffraction network section, we studied the effects of target shape and area on the output spectrum, and explored the effects of the relative position of the scattering layer on the output spectrum. The results show that the network can respond well to changes in target shape and is insensitive to errors in the relative position of components. During the training process of the entire network, we used a total of 300 sample data, and finally completed the training of the network in 9.2 s under low hardware conditions, achieving a classification accuracy of 93.3%. The results show that this opto-electronic hybrid network can quickly complete training under low hardware conditions and has high accuracy. This research has application value in the fields of target detection, machine vision and turbulence intensity measurement.

## Figures and Tables

**Table 1:** Time and accuracy comparison of the three networks in various scenarios.

Category	Data Size	Time	Accuracy Rate	Hardware
CNN (3 classes) [37]	2400	Long time	99.74%	Ubuntu 16.04 with a GTX 1080 GPU
CNN (14 classes) [38]	200	67 h	99%	I7
Optical diffraction network1 (2 classes) [24]	12,500	5 h	99.53%	I9-7900X with RTX 3090
Optical diffraction network2 (10 classes) [39]	8000	20 h	84%	E5-2650 with RTX 1080Ti
Opto-electronic hybrid network (3 classes)	100	9.2 s	93.3%	I5-7300H with RTX 1050Ti

## Data Availability

The data are contained within the article.

## References

[1] Ball J., Anderson D., Chan C.S. (2017). Comprehensive survey of deep learning in remote sensing: Theories, tools, and challenges for the community. J. Appl. Remote Sens..

[2] Tian L., Fan C., Ming Y. (2016). Multiple scales combined principle component analysis deep learning network for face recognition. J. Electron. Imaging.

[3] Berco D., Shenp Ang D. (2019). Recent Progress in Synaptic Devices Paving the Way toward an Artificial Cogni-Retina for Bionic and Machine Vision. Advanced Intelligent Systems.

[4] Zhang X., Gu N., Ye H., Lin C. (2018). Vehicle license plate detection and recognition using deep neural networks and generative adversarial networks. J. Electron. Imaging.

[5] Shi W., Huang Z., Huang H., Hu C., Chen M., Yang S., Chen H. (2022). LOEN: Lensless opto-electronic neural network empowered machine vision. Light Sci. Appl..

[6] Luo Y., Yan S., Li H., Lai P., Zheng Y. (2020). Focusing light through scattering media by reinforced hybrid algorithms. APL Photonics.

[7] Li Y., Xiong D., Zhang M. (2018). A Survey of Neural Machine Translation. Chin. J. Comput..

[8] Zhang S., Wang H., Chen P., Zhang X., Li Q. (2022). Overview of the application of neural networks in the motion control of unmanned vehicles. Chin. J. Eng..

[9] Yoon H.J., Kim S., Kim J.-H., Keum J.-S., Oh S.-I., Jo J., Chun J., Youn Y.H., Park H., Kwon I.G. (2019). A Lesion-Based Convolutional Neural Network Improves Endoscopic Detection and Depth Prediction of Early Gastric Cancer. J. Clin. Med..

[10] Melzer R., Severa W., Vineyard C. (2022). Exploring SAR ATR with neural networks: Going beyond accuracy. Proceedings of the Automatic Target Recognition XXXII.

[11] Chen G., Wei S. (2022). Fusion sampling networks for skeleton-based human action recognition. J. Electron. Imaging.

[12] Tong X., Sun S., Fu M. (2021). Disentangled-region non-local neural network for facial expression recognition. J. Electron. Imaging.

[13] Jia P., Ma M., Cai D., Wang W., Li J., Li C. (2021). Compressive Shack–Hartmann wavefront sensor based on deep neural networks. Mon. Not. R. Astron. Soc..

[14] Wong A., Norris B.R., Tuthill P., Scalzo R., Lozi J., Vievard S., Guyon O. (2021). Predictive control for adaptive optics using neural networks. J. Astron. Telesc. Instrum. Syst..

[15] Andersen T., Owner-Petersen M., Enmark A. (2020). Image-based wavefront sensing for astronomy using neural networks. J. Astron. Telesc. Instrum. Syst..

[16] Chen X. (2021). Research on Improving Generalization Ability Based on LSTM Network.

[17] Pappas C., Moschos T., Alexoudi T., Vagionas C., Pleros N. (2023). 16-Bit (4 × 4) Optical Random Access Memory (RAM) Bank. J. Light. Technol..

[18] Spall J., Guo X., Barrett T.D., Lvovsky A.I. (2020). Fully reconfigurable coherent optical vector–matrix multiplication. Opt. Lett..

[19] Robertson J., Kirkland P., Alanis J.A., Hejda M., Bueno J., Di Caterina G., Hurtado A. (2022). Ultrafast neuromorphic photonic image processing with a VCSEL neuron. Sci. Rep..

[20] Chen B., Zhang Z., Dai T., Yu H., Wang Y., Yang J. (2023). Photonic Neural Networks and Its Applications. Laser Optoelectron. Prog..

[21] Hattori N., Masuda Y., Ishihara T., Shiomi J., Shinya A., Notomi M. (2021). Optical-Electronic Implementation of Artificial Neural Network for Ultrafast and Accurate Inference Processing. Proceedings of the AI and Optical Data Sciences II.

[22] Pankov A.V., Vatnik I.D., Sukhorukov A.A. (2022). Optical Neural Network Based on Synthetic Nonlinear Photonic Lattices. Phys. Rev. Appl..

[23] Li Q., Zhao J., Zhang Y., Lai X., Chen Z., Pu J. (2020). Imaging reconstruction through strongly scattering media by using convolutional neural networks. Opt. Commun..

[24] Bai B., Li Y., Luo Y., Li X., Çetintaş E., Jarrahi M., Ozcan A. (2023). All-optical image classification through unknown random diffusers using a single-pixel diffractive network. Light Sci. Appl..

[25] Mengu D., Tabassum A., Jarrahi M., Ozcan A. (2023). Snapshot multispectral imaging using a diffractive optical network. Light Sci. Appl..

[26] Zhou T., Fang L., Yan T., Wu J., Li Y., Fan J., Wu H., Lin X., Dai Q. (2020). Optical backpropagation training method and its applications. Proceedings of the Optoelectronic Imaging and Multimedia Technology VII.

[27] Li J., Mengu D., Luo Y., Rivenson Y., Ozcan A. (2019). Class-specific differential detection in diffractive optical neural networks improves inference accuracy. Adv. Photonics.

[28] Vellekoop I.M., Lagendijk A., Mosk A.P. (2010). Exploiting disorder for perfect focusing. Nat. Photonics.

[29] Wang B., Zheng M.-Y., Han J.-J., Huang X., Xie X.-P., Xu F., Zhang Q., Pan J.-W. (2021). Non-Line-of-Sight Imaging with Picosecond Temporal Resolution. Phys. Rev. Lett..

[30] Sahoo S.K., Tang D., Dang C. (2017). Single-shot multispectral imaging with a monochromatic camera. Optica.

[31] Schott S., Bertolotti J., Léger J.-F., Bourdieu L., Gigan S. (2015). Characterization of the angular memory effect of scattered light in biological tissues. Opt. Express.

[32] Yılmaz H., Kühmayer M., Hsu C.W., Rotter S., Cao H. (2021). Customizing the Angular Memory Effect for Scattering Media. Phys. Rev. X.

[33] Shi Y., Guo E., Sun M., Bai L., Han J. (2022). Non-invasive imaging through scattering medium and around corners beyond 3D memory effect. Opt. Lett..

[34] Kohlgraf-Owens T., Dogariu A. (2008). Finding the field transfer matrix of scattering media. Opt. Express.

[35] Loran F., Mostafazadeh A. (2016). Transfer matrix formulation of scattering theory in two and three dimensions. Phys. Rev. A.

[36] Kim M., Choi W., Choi Y., Yoon C., Choi W. (2015). Transmission matrix of a scattering medium and its applications in biophotonics. Opt. Express.

[37] Wen L., Li X., Gao L., Zhang Y. (2018). A New Convolutional Neural Network-Based Data-Driven Fault Diagnosis Method. IEEE Trans. Ind. Electron..

[38] Gulzar Y., Hamid Y., Soomro A.B., Alwan A.A., Journaux L. (2020). A Convolution Neural Network-Based Seed Classification System. Symmetry.

[39] Chen H., Feng J., Jiang M., Wang Y., Lin J., Tan J., Jin P. (2021). Diffractive Deep Neural Networks at Visible Wavelengths. Engineering.

